# Automating sentinel-1 SLC product processing: Parallelization and optimization for efficient polarimetric parameter extraction

**DOI:** 10.1016/j.mex.2025.103253

**Published:** 2025-03-04

**Authors:** Hansanee Fernando, Kwabena Nketia, Thuan Ha, Sarah van Steenbergen, Heather McNairn, Steve Shirtliffe

**Affiliations:** a Department of Plant Sciences, College of Agriculture and Bioresources, University of Saskatchewan, Canada; b Agriculture and Agri-Food Canada, Ottawa, Canada

**Keywords:** Automation, Sentinel-1, SNAP, PolSARpro, Parallelization, RStudio, Batch-processing, Single Look Complex, Automation of processing S1 SLC images

## Abstract

Processing Sentinel-1 (S1) Single Look Complex (SLC) data is time-consuming, even with software like SNAP or PolSARpro. Command line processing on Windows provides an automated alternative, enabling R-based processing of multiple S1-SLC files without manual interaction. Here we demonstrate a user friendly automated process, to process an unlimited number of S1-SLC images, tailored for users with minimal SAR or programming competence. The proposed workflow integrates RStudio, SNAP, and PolSARpro software libraries to implement the same processes a user can achieve via the corresponding graphic user interfaces (GUI). The workflow includes bulk S1-SLC imagery downloads, installation and configuration of dependent software applications. Within the SNAP GUI, a base-graph was constructed, encompassing crucial processing steps such as data import, sub-swath extraction, orbit determination, calibration, speckle filtering, debursting, and terrain correction, which acts as a template for generating customized SNAP graphs for individual S1 imagery. These graphs are batch processed with R, using parallel computing to run multiple graphs simultaneously. In the subsequent PolSARpro processing phase, outputs from the SNAP processing pipeline are made interoperable with PolSARpro tools for onward post-processing. Similarly, we leverage the parallelization mechanisms of R for user specific parameter extraction, which maximizes resource utilization while maintaining computational performance.

• Automated Workflow for SAR Processing: Introduces an automated, user-friendly framework combining RStudio, SNAP, and PolSARpro to process unlimited Sentinel-1 Single Look Complex (S1-SLC) images, eliminating manual interaction and catering to users with minimal programming or SAR expertise.
• Customizable and Scalable Processing: Leverages SNAP's base-graph templates for essential SAR processing steps (e.g., orbit determination, calibration, speckle filtering, and terrain correction) to enable batch processing and parallel computing for efficient handling of large datasets.
• Interoperability and Enhanced Performance: Integrates outputs from SNAP into PolSARpro for advanced post-processing, employing R-based parallelization to optimize resource utilization and ensure efficient user-specific parameter extraction.

## Graphical abstract

**Figure:**
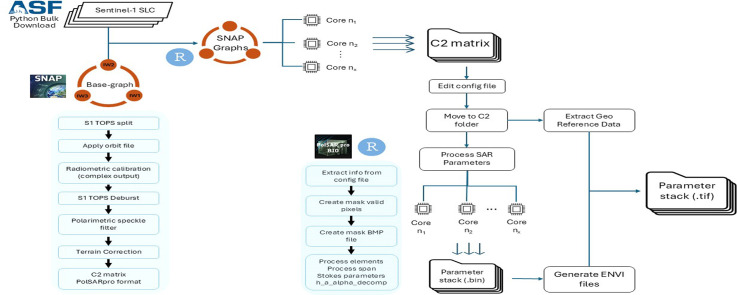

Specifications table

**Table:** 

Subject area:	Earth and Planetary Sciences
More specific subject area:	*Remote Sensing*
Name of your method:	*Automation of processing S1 SLC images*
Name and reference of original method:	*ARSET. (2022). Mapping Crops and their Biophysical Characteristics with Polarimetric SAR and Optical Remote Sensing. NASA Applied Remote Sensing Training Program (ARSET).*
Resource availability:	https://github.com/Smf566/S1_SNAP_Polsar

## Background

The growing reliance on Sentinel-1 (S1) Synthetic Aperture Radar (SAR) data for Earth observation applications, including agriculture, sea ice monitoring, and natural hazard assessment, underscores its value in scientific research and practical applications [5,7]. Among its operational modes, the Interferometric Wide (IW) swath mode is extensively utilized for land and coastal monitoring due to its ability to acquire data in three sub-swaths (IW1, IW2, IW3) with multiple bursts, offering consistent phase information critical for interferometry and polarimetric analysis [4,10]. However, leveraging the full potential of S1 SAR data—particularly Slant Range Single Look Complex (SLC) products—remains a challenge due to the extensive expertise and computational resources required for processing.

S1-SLC data is uniquely suited for detailed environmental applications due to its high spatial resolution and retention of both amplitude and phase information, enabling advanced analyses such as interferometry and polarimetry [9]. For instance, S1 intensity data has achieved significant success, such as in-season crop mapping with an accuracy of 80.3 % [1]. However, the absence of phase data in intensity products limits their application, emphasizing the need for SLC data to unlock advanced capabilities. Despite this, processing SLC data involves complex steps like generating the C2 covariance matrix—a critical element for polarimetric SAR analysis—making it accessible primarily to those with specialized expertise [2].

The challenges extend further to the limitations of existing software tools like SNAP and PolSARpro, which, while powerful, rely on manual workflows. Although command-line processing options offer greater efficiency, the lack of comprehensive online resources and standard operating protocols hinders their adoption among users with limited programming skills [8]. This inefficiency in handling large volumes of data across diverse spatial extents has become a significant bottleneck for researchers and practitioners alike.

To address these challenges, this study introduces a streamlined workflow integrating SNAP and PolSARpro with RStudio. By leveraging the parallelization capabilities of R, the proposed methodology automates key aspects of SLC data processing, significantly reducing the manual effort required. This innovation democratizes access to advanced polarimetric parameters, such as decomposition techniques and Stokes parameters, enabling their application in critical environmental studies like crop phenological analysis [3].

The workflow not only simplifies the extraction of essential polarimetric parameters but also enhances scalability, making it an invaluable resource for users with limited SAR expertise or programming competence. By bridging the gap between advanced SAR data processing and user accessibility, this methodology fosters the wider adoption of S1-SLC data in environmental sciences, empowering researchers to unlock its full potential in a diverse range of applications.

## Method details

Detailed workflow, which includes comments and itemized stages that were used for setting up and configuring SNAP, RStudio, and PolSARpro are provided in the R script and the readme file available on a cloud-optimized repository (https://github.com/Smf566/S1_SNAP_Polsar).

Step 1: Installation and Configuration of Software

This outlines the installation and configuration of essential software tools for processing S1 SLC data, including Python, RStudio, SNAP, and PolSARpro. Python and RStudio installations facilitate data download and parallelized processing, while SNAP provides the necessary tools for generating the C2 matrix. PolSARpro is used for advanced polarimetric data processing. Configuration steps include adding relevant directories to the system PATH environment variable, ensuring that command-line interfaces can access the executables directly. Verification steps are included to confirm successful installations, ensuring that each tool is ready for integration into the processing workflow.

Python and RStudio for Data Download and Parallelized Processing

• Python (Latest Stable Version: 3.12.1, Released: January 2024)
• R (Latest Stable Version: 4.3.2, Released: February 2024) & RStudio

SNAP (Sentinel Application Platform) for C2 Matrix Generation

• Latest Version: SNAP 11.0.0 (Released: October 24, 2024, 13:00 UTC)
• Download: The Sentinel Toolboxes compatible with Windows 64-bit can be downloaded from the ESA SNAP official website.

PolSARpro for Advanced Polarimetric Processing

• Latest Version: PolSARpro v6.0 (Biomass Edition), Version 6.0.4 (Released: January 22, 2025)

Configuration and Environment Setup

To ensure smooth integration of these tools into the processing workflow, follow these configuration steps:

• Add the installation directories of Python, R, SNAP, and PolSARpro to the system PATH environment variable.
• Verify successful installation by running version-check commands in the command line:

python –version (Expected output: Python 3.12.1)

R –version (Expected output: R 4.3.2) gpt –version (For SNAP command-line tools)

PolSARpro (To check the software startup)

Step 2: Bulk Download of Sentinel-1 SLC Images

**Fig. 1:**
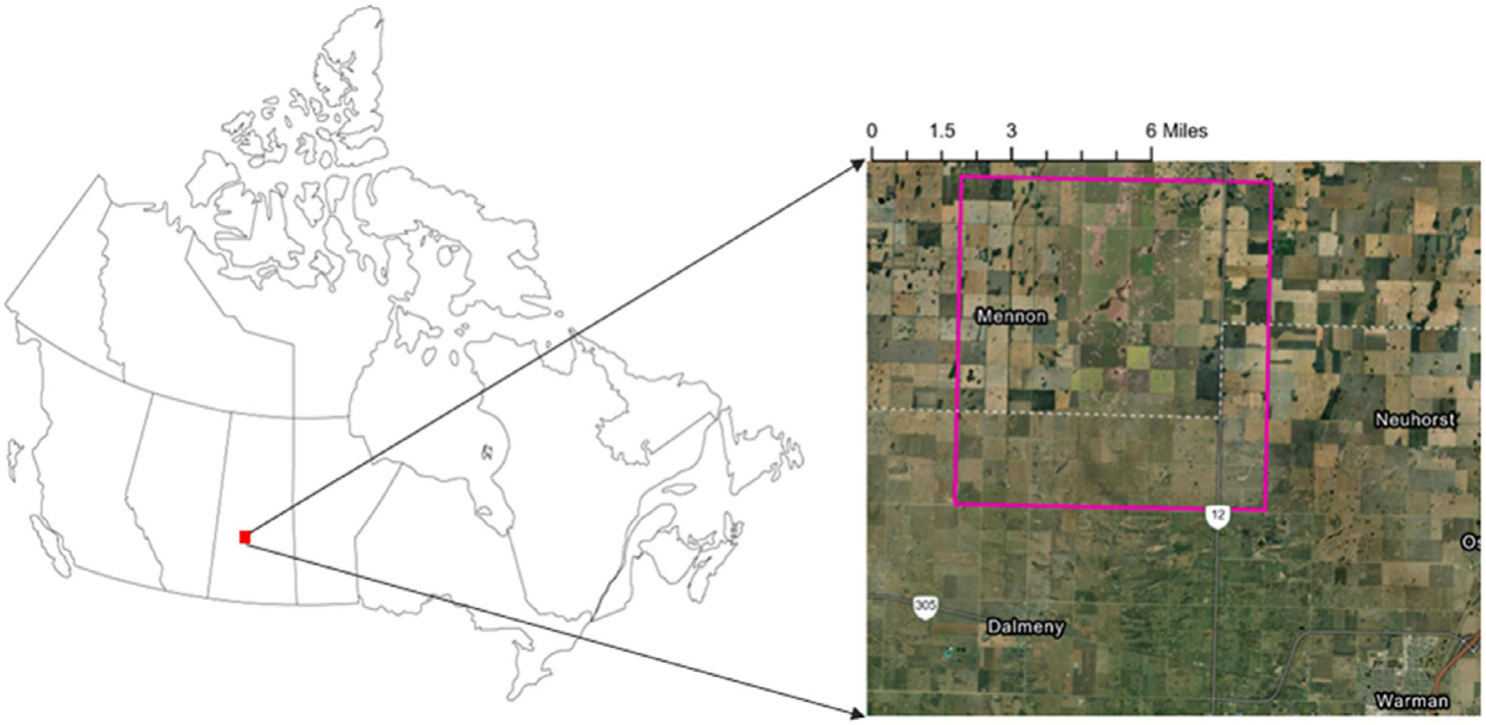
Study location,Saskatoon, Saskactchewan, Canada.

S1-SLC images were acquired using the Alaska Satellite Facility (ASF) Data Search platform. Users of the ASF platform need an Earth Data account to access and download S1 imagery. See (ASF, 2024) for bulk download instructions. In the present use case demonstrated in this paper, S1-SLC imagery collection (total of 9 images) were obtained from June 10, 2021, to July 30, 2021, for the test study area (Fig. 1). To ensure commonality across the proposed workflow, image collection was downloaded using the Python script, which was auto generated on the Earth Data account platform and subsequently implemented via a local command prompt interface. See the attached readme file in the supplementary material.

Step 3: SNAP Processing (within R)
Step 3.0 Generate the base graph

In the SNAP processing phase, the initial step involves generating the base-graph, which encompasses importing a single S1 image in zip file format into the SNAP software interface. A sample graph is then constructed with a sequence of processing methods in the correct order [6]. The process begins with data importation using the ``Read'' tool, followed by sub-swath extraction using the ``TOPSAR-Split'' tool. Subsequently, precise orbit determination is conducted utilizing the ``Apply-Orbit- File'' tool, followed by data calibration with the ``Calibration'' tool, and debursting using the ``TOPSARDeburst'' tool. Further refinement is achieved through polarization speckle filtering facilitated by the ``Polarimetric-Speckle-Filter'' tool. Geometric distortions are corrected using the ``Terrain-Correction'' tool, ensuring accurate spatial representation. Finally, the processed data is exported to PolSARpro format using the ``Write'' tool, enabling seamless integration with downstream analysis tools. A base-graph is provided in the supplementary material and users are encouraged to configure the parameters of each tool according to their specific preferences and requirements. To accommodate different sub-swaths, three graphs are generated for each subswath (IW1, IW2, and IW3).

Step 3.1 Preparing the directories and the base-graph for processing

In this stage, the necessary directories and base files for processing S1 images were established. The `xml2` package in R was utilized to identify and modify the base SNAP graph file. Interferometric Wide (IW) swaths were also selected for processing. This preparation ensures an organized and systematic approach to managing and processing the extensive datasets used in polarimetric synthetic aperture radar (PolSAR) analysis.

Step 3.2 Generate graphs for each S1 file

This stage involves generating corresponding graphs (.xml) for each S1 zip file. This process leads to the creation of 27 SNAP graphs, representing each combination of the nine zip files and three IW swaths. The uniformity is maintained across all SNAP graphs, with adjustments made to input and output names as necessary. Leveraging the base-graph facilitates the efficient generation of tailored SNAP graphs for individual S1 files, streamlining the overall workflow.

Step 3.3 Batch processing of SNAP graphs

**Fig. 2:**
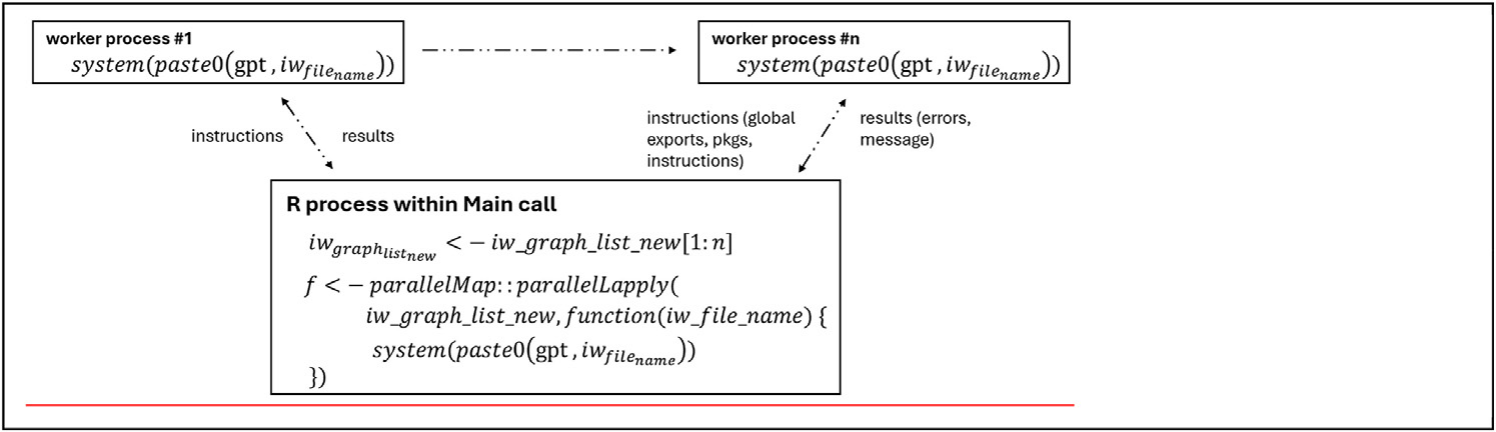
R parallelization.

In the batch processing stage, the computational capabilities of the system are leveraged to execute multiple graphs concurrently within R. By assigning an optimal number of cores (e.g., *n* = 3), the library ‘parallelMap’ (Bischl et al., 2021) in R is employed to process three graphs simultaneously (Fig. 2). Experimentation with the number of available cores is recommended, monitoring memory and CPU usage to determine the optimal configuration. This approach maximizes processing speed while maintaining optimal memory and CPU performance. This parallel processing approach maximizes computational efficiency and minimizes processing time, effectively utilizing the available computing resources.

Step 4: PolSARpro Processing (within R)
Step 4.1 Data configuration

In the PolSARpro processing phase, data preparation and configuration are essential initial steps. The SNAP software outputs C2 matrix files in the form of .bin and .hdr files, which are indispensable for PolSARpro processing. To ensure compatibility, the configuration file, config.txt, requires editing to replace the parameter ``dual'' with ``pp2''. This adjustment allows PolSARpro to properly recognize and process the data.

Step 4.2 Generation of C2 folder and preserving geo-spatial information

All relevant files are organized into a directory named C2. This adjustment allows PolSARpro to properly recognize and process the data. Additionally, the .hdr file generated by SNAP contains geospatial information specific to the corresponding IW swath. Utilizing C11.hdr, a reference tiff file is created to preserve this geospatial information, facilitating accurate spatial representation throughout the processing pipeline.

Step 4.3 Generating PolSARpro parameters

Configuration File Details

The config.txt file plays a crucial role in PolSARpro processing, containing metadata such as the number of rows and columns essential for subsequent parameter generation. This metadata guides the processing workflow within PolSARpro, ensuring accurate and efficient processing.

Mask Creation and Validation

The next phase involves mask creation and validation, where valid mask pixels are identified using the create_mask_valid_pixels tool. This mask ensures that only valid data points are utilized in further processing, enhancing the accuracy and reliability of the results.

Parameter generation

The next phase involves developing essential decomposition parameters using various PolSARpro tools. To enhance efficiency, this process can be parallelized within R, leveraging the computer's computational capacity.

*Generation of the C2 covariance matrix*

The first step in the polarimetric decomposition involves constructing the C2 covariance matrix from the dual-polarized Sentinel-1 data using SNAP (up to step 4.2). The C2 matrix is a reduced form of the full polarimetric covariance matrix (C3 or C4) and is given by

$$C2\;=\;\left[\begin{array}{cc} C_{11} & C_{12}\\ C_{21} & C_{22} \end{array}\right]$$

Initially, the C2 elements are developed using the `process_elements` tool, forming the basis for subsequent parameter calculations. where:

• C_11_ = ∣S_VV_∣^2^ = Backscatter intensity for VV channel
• C_22_ = ∣S_VH_∣^2^ = (Backscatter intensity for VH channel)
• C_12_ = $S_{\text{VV}}S_{\text{VH}}^{*}$ = (Cross-correlation term)
• C_21_ = $S_{\text{VH}}S_{\text{VV}}^{*}$ = (Complex conjugate of C12)

The total span, representing the sum of the backscattered power, was derived using the `process_span` tool. The span provides insights into the intensity of the scattered signal and is calculated as:

• Span = C_11_ + C_22_ = |S_VV_|² + |S_VH_|²

The Stokes parameters (g_0_, g_1_, g_2_, g_3_) describe the polarization characteristics of the SAR signal and are computed using the `stokes_parameters` tool. The Stokes parameters are defined as follows:

• g_0_ = |S_VV_|² + |S_VH_|² = Total intensity
• g_1_ = |S_VV_|² - |S_VH_|² = Difference in linear polarization intensities
• g_2_ = 2Re(S_VV_ S_VH_*) = Preponderance of linear +45° polarized light over linear −45° polarized light
• g_3_ = 2Im(S_VV_ S_VH_*) = Preponderance of right-handed circular polarized light over left-handed circular polarized light

The ‘stokes_parameters’ tool allow the computation of Stokes angles, which describe the polarization ellipse of the scattered wave:

• Orientation angle (ψ)
  $$\psi \;=\;\frac{1}{2}\;tan^{-1}\left(\frac{g_{2}}{g_{1}}\right)$$
• Ellipticity angle (χ)
  $$\chi \;=\;\frac{1}{2}\;tan^{-1}\left(\frac{g_{3}}{g_{0}}\right)$$

Wave descriptors, extracted using the `stokes_parameters` tool, provide additional insights into the coherence and polarization purity of the observed target:

• Eigenvalues of the covariance matrix = λ1, λ2: Represents dominant scattering components
• Degree of Linear Polarization (DoLP): proportion of the signal that is linearly polarized
  $$\text{DoLP}\;=\;\frac{\sqrt{g_{1}^{2}+g_{2}^{2}}}{g_{0}}$$
• Linear Polarization Ratio (LPR): distinguish between different scattering mechanisms
  $$\text{LPR}\;=\;\frac{{\left|S_{VV}\right|}^{2}}{{\left|S_{VH}\right|}^{2}}$$

The `h_a_alpha_decompositionSPPC2` tool is utilized to compute Alpha, Entropy and Shannon Entropy

• Entropy (H): Measures the randomness of the scattering process
  $$H\;=\;-\sum p_{i}Log_{2}p_{i}$$

where p_i_ are eigenvalue-derived probabilities.

• Alpha angle (α): Represents the dominant scattering mechanism
  $$\alpha =\sum p_{i}\alpha_{i}$$
• Shannon Entropy: Provides information entropy of the scattering process, indicating the amount of uncertainty present in the target backscatter

Step 4.4 ENVI file generation

The .hdr file output from the SNAP process contains crucial geospatial information specific to each individual IW swath. Only the parameters generated through SNAP will have corresponding .hdr files (C11, C12_imag, C12_real, C22). To process the images to TIFF files, each file must be associated with .hdr files with the map information that stores the geospatial coordinates. Parameters generated through PolSARpro will only create .bin files. To create corresponding .hdr files, C11.hdr file was used as a reference. This process involves extracting relevant spatial metadata from the C11.hdr file and formatting it into an ENVI-compatible format.

Step 4.5 Rasterization

Raster files play a pivotal role in visualizing and interpreting processed SAR data within a geographic context. These files, derived from the extracted spatial information contained within the previously generated .hdr files, preserve the accuracy of geospatial data. By utilizing this spatial information, the generated raster files ensure that the processed SAR data maintains its spatial integrity, allowing users to visualize and analyze the data within the framework of its geographic location. This spatial accuracy is crucial for interpreting SAR data accurately, as it enables users to identify and analyze features and phenomena within their real-world geographical context. Thus, the creation of raster files facilitates comprehensive and meaningful analysis of SAR data, enhancing its utility for a wide range of applications, including environmental monitoring, disaster management, and land use planning.

## Limitations

Not applicable.

## Ethics statements

This study does not involve human subjects, animal experiments, or the use of data from social media platforms.

## CRediT authorship contribution statement

**Hansanee Fernando:** Writing – original draft, Conceptualization, Methodology. **Kwabena Nketia:** Conceptualization, Methodology, Writing – review & editing. **Thuan Ha:** Validation. **Sarah van Steenbergen:** Validation. **Heather McNairn:** Writing – review & editing. **Steve Shirtliffe:** Writing – review & editing, Supervision.

## Declaration of competing interest

The authors declare that they have no known competing financial interests or personal relationships that could have appeared to influence the work reported in this paper.

## Data Availability

All codes will be provided in GitHub Page.

## Appendix Supplementary materials

Image, application 1

Image, application 2
